# Supplementary material for: The Role of Preservation Solutions upon Saphenous Vein Endothelial Integrity and Function: Systematic Review and UK Practice Survey
Source: Cells. 2023 Mar 6;12(5):815. doi: 10.3390/cells12050815 (PMC10001248; doi:10.3390/cells12050815)
Supplement: Supplementary file 1 [file cells-12-00815-s001.zip › cells-2231582-supplementary.pdf]

Supplementary Table S1. Literature search strategy.

<u>Ovid MEDLINE(R) ALL <1946 to August 09, 2022></u>

1 exp Coronary Artery Bypass/

2 (Coronary Artery Bypass or CABG).mp.

3 Saphenous Vein.mp.

4 exp Saphenous Vein/

5 (preservation solution or storage solution or saline solution or heparinised blood or buffered solution).mp.

6 (AHB or GALA or HTK or UWS).mp.

7 (University of Wisconsin or Histidine-tryptophan-ketoglutarate or TiProtec or He solution or Glutathione-ascorbic L-arginin).mp.

8 1 or 2

9 3 or 4

10 5 or 6 or 7

11 8 and 9 and 10

Total = **46**

<u>Embase <1974 to 2022 August 09></u>

1 exp Coronary Artery Bypass/

2 (Coronary Artery Bypass or CABG).mp.

3 Saphenous Vein.mp.

4 exp Saphenous Vein/

5 (preservation solution or storage solution or saline solution or heparinised blood or buffered solution).mp.

6 (AHB or GALA or HTK or UWS).mp.

7 (University of Wisconsin or Histidine-tryptophan-ketoglutarate or TiProtec or He solution or Glutathione-ascorbic L-arginin).mp.

8 1 or 2

9 3 or 4

10 5 or 6 or 7

11 8 and 9 and 10

Total = **75**

Web Of Science through Clarivate (09/08/22)

((ALL=(((Saphenous Vein) OR (GSV)))) AND ALL=((Coronary Artery Bypass OR CABG))) AND ALL=(preservation solution or storage solution or saline solution or heparinised blood or buffered solution or AHB or GALA or HTK or UWS or University of Wisconsin or Histidine-tryptophan-ketoglutarate or TiProtec or He solution or Glutathione-ascorbic L-arginin)

Total = **101**
